# Wool Keratin-Associated Protein Genes in Sheep—A Review

**DOI:** 10.3390/genes7060024

**Published:** 2016-05-28

**Authors:** Hua Gong, Huitong Zhou, Rachel H. J. Forrest, Shaobin Li, Jiqing Wang, Jolon M. Dyer, Yuzhu Luo, Jon G. H. Hickford

**Affiliations:** 1 International Wool Research Institute, Faculty of Animal Science and Technology, Gansu Agricultural University, Lanzhou 730070, China; gonghua3000@gmail.com (H.G.); zhouh@lincoln.ac.nz (H.Z.); lisb@gsau.edu.cn (S.L.); wangjq@gsau.edu.cn (J.W.); 2 Gene-marker Laboratory, Faculty of Agricultural and Life Sciences, Lincoln University, Lincoln 7647, New Zealand; 3 Faculty of Health and Sport Sciences, Eastern Institute of Technology, Private Bag 1201, Napier 4142, New Zealand; rforrest@eit.ac.nz; 4 Food & Bio-Based Products, Lincoln Research Centre, AgResearch Limited, Lincoln 7608, New Zealand; dyerj@agresearch.co.nz

**Keywords:** Keratin-associated protein (KAP), polymorphism, expression, wool fibre, wool traits, sheep

## Abstract

The importance of sheep’s wool in making textiles has inspired extensive research into its structure and the underlying genetics since the 1960s. Wool keratin-associated proteins (KAPs) are a key structural component of the wool fibre. The characterisation of the genes encoding these proteins has progressed rapidly with advances in the nucleotide and protein sequencing. This review describes our knowledge of ovine KAPs, their categorisation into families, polymorphism in the proteins and genes, the clustering and chromosomal location of the genes, some characteristics of gene expression and some potential effects of the KAPs on wool traits. The extent and nature of genetic variation in wool KAP genes and its association with fibre characteristics, provides an opportunity for the development of gene-markers for selective breeding of sheep to produce better wool with properties highly matched to specific end-uses.

## 1. Introduction

Wool is a natural fibre with unique attributes. It is widely used in the apparel, insulation and carpet industries, and humans have long recognised the benefits of wool, including its thermal attributes, breathability and fire resistance [1].

Wool fibre is primarily composed of proteins called hard α-keratins [2]. These have a high sulphur content, consistent with having a high relative abundance of the amino acid cysteine. In wool, the α-keratins are assembled into keratin intermediate filaments (KIFs), before being embedded in an inter-filamentous matrix containing keratin-associated proteins (KAPs). There are also other protein components in wool, such as trichohyalin, which is located in the inner root sheath and the medulla of the fibre, but these proteins are not regarded as essential to the fibre structure [2].

The earliest attempt to identify and classify wool proteins was made in 1935 [3], and it divided the major wool components into two classes of extractable protein: S-carboxy methyl kerateine A (SCMK-A) and S-carboxy methyl kerateine B (SCMK-B). These were of lower and higher sulphur content than the average sulphur content of wool respectively, and subsequently the SCMK-As were found to be the hair and wool α-keratins, while the SCMK-Bs were the KAPs [4].

KAPs interact with the KIFs during fibre development and covalently bind with the KIFs through extensive disulphide bond cross-linking between cysteine in the KAPs and in the head and tail domains of the keratins [5]. Bundles of KIFs, then combine with KAPs to form macrofibrils through further inter- and/or intra-molecular disulphide bond formation [6]. While the KAPs may have little, or no discernible effect on keratin IF structure, their effect on KIF assembly into larger arrays, is considered to be crucial [7]. It is therefore believed that KAPs play an important role in defining the physico-mechanical properties of the wool fibre.

The KAPs are small in size (*ca.* 10–30 kDa) and while they typically possess a high cysteine content, they can also have a high glycine and tyrosine content [5,8].

## 2. The Complexity of Wool KAPs

Electrophoretic analyses of the SCMK-Bs revealed that a large number of KAPs existed [5]. Seven KAPs with a high sulphur content (B2A or KAP1-1, B2B or KAP1-2, B2C or KAP1-3, BIIIA3 or KAP2-n, BIIIB2 or KAP3-1, BIIIB3 or KAP3-3 and BIIIB4 or KAP3-3) were initially isolated from wool and their amino acid sequences were determined [9,10,11,12,13,14,15,16] and two KAPs with a high glycine-tyrosine content were also isolated and characterised [17,18].

With the advent of DNA cloning techniques, 16 gene sequences encoding KAPs of a high sulphur content [19,20,21,22,23] and a high glycine-tyrosine content [24,25,26] were discovered through the isolation of their cDNA or genomic sequences. The subsequent development of further gene technologies, together with the sequence information derived from other species, has led to the identification of another five high sulphur KAP genes [27,28,29,30,31] and four high glycine-tyrosine [32,33,34] KAP genes in sheep.

Sequence analyses suggest that the KAP sequences identified to date probably represent 27 different KAP family members (Table 1). However, the possibility cannot be ruled out that some of these 27 may represent different variants of the same gene, rather than being different family members. This was shown for the human KAP1 [35] and KAP4 [36] genes. This suggests that further investigation is required to confirm both the number and constituency of the families, especially as our current understanding of the wool KAPs is still limited. For example, there may be ovine KAP genes that have not been identified yet, especially given that 88 functional and 17 pseudo-KAP genes have now been described in the human genome [37,38,39]. This likely represents the complete number of KAPs in humans, although sequencing of more human genetic material from different races and genetic backgrounds may result in minor changes to this thinking.

## 3. The Categorisation and Classification of Wool KAPs

Following the original classification into SCMK-A and SCMK-B, the KAPs were subsequently categorised into three groups according to their amino acid composition: the high sulphur (HS; ≤30 mol% cysteine), the ultra-high sulphur (UHS; >30 mol% cysteine) and the high glycine/tyrosine (HGT; 35–60 mol% glycine and tyrosine) groups [5]. They were then assigned into families based on amino acid sequence homology.

In general terms, the individual KAP families are distinguished by the cysteine, or glycine and tyrosine content, the type and number of amino acid repeat structures, and the occurrence of unique sequence motifs [8]. In humans, the 88 functional KAP genes identified have been assigned to 25 families: KAP1 to KAP27, but with the absence of KAP14 and KAP18, which have only been found in mice [37,38,39].

**Figure 1:**
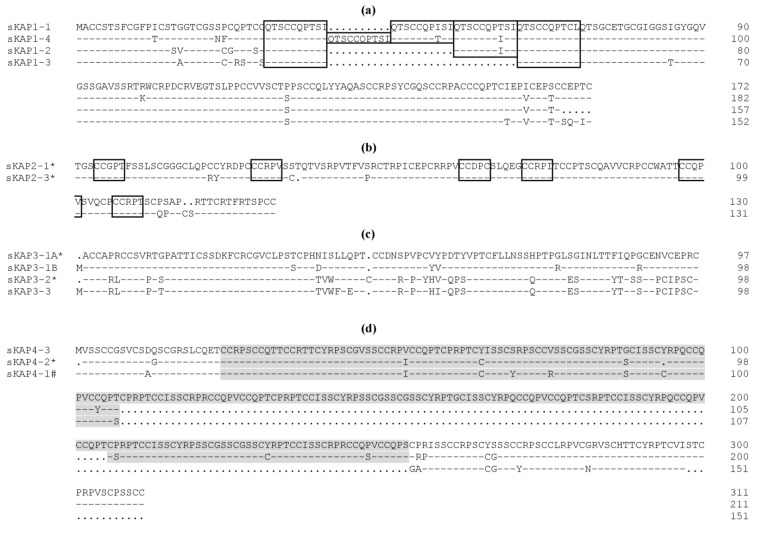

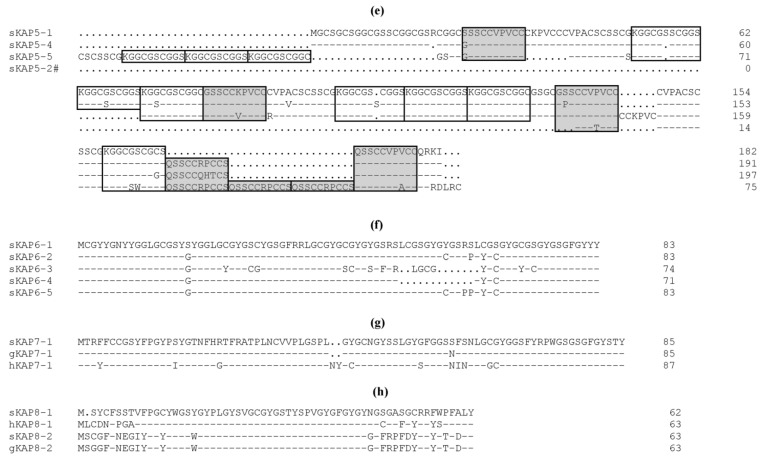

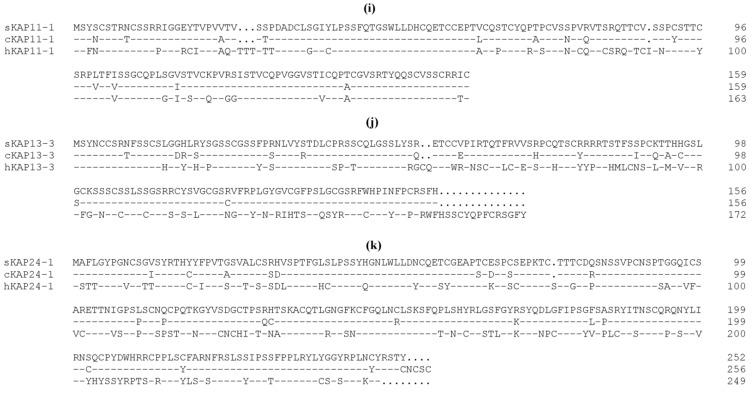
Amino acid sequence alignments of representative sequences from the ovine KAP families and orthologous sequences from other species. (**a**) KAP1-n; (**b**) KAP2-n; (**c**) KAP3-n; (**d**) KAP4-n; (**e**) KAP5-n; (**f**) KAP6-n; (**g**) KAP7-1; (**h**) KAP8-n; (**i**) KAP11-1; (**j**) KAP13-3; and (**k**) KAP24-1. The dashes represent amino acids identical to the top sequence and the dots act as spacers to improve alignments. The QTSCCQPXXX decapeptide repeat sequence found in KAP1-n and the CCVPX pentameric repeat sequence found in KAP2-n are shown in boxes. The central region consisting of contcatenates of nonocysteine- and dicysteine-containing pentameric repeats found in KAP4-n is shaded. In KAP5-n, the glycine-rich decapeptide repeat sequence is boxed, whereas the cycteine/serine-rich decapeptide repeat sequence is both boxed and shaded. All the amino acid sequences are predicated from DNA sequences, except for those indicated by * which were derived directly from protein sequencing. # indicates a partial sequence. The sheep, goat, cattle and human sequences are indicated with a prefix of “s”, “g”, “c” and “h”, respectively. Accession numbers or references for the ovine sequences are shown in Table 1, and those from other species are: AB096962 (hKAP7-1); AY510121 (gKAP7-1); AP001709 (hKAP8-1); AY510123 (gKAP8-2); NM_001080740 (cKAP11-1); NW_001838706 (hKAP11-1); ENSBTAG00000040032 (cKAP13-3); NM_181622.1 (hKAP13-3); XM_002684598.1 (cKAP24-1) and NM_001085455 (hKAP24-1).

In sheep, 27 putative wool KAPs having been identified to date and these have been assigned into the following 11 families:

**KAP1**: KAP1 is a HS-KAP family that contains four members (KAP1-1, KAP1-2, KAP1-3 and KAP1-4) [13,14,19,29]. The number of members equals that described in humans [8,40]. The KAP1 family members are similar to each other and differ mainly in the number of conserved tandem decapeptide “QTSCCQPXXX” repeats in the N-terminal half of the proteins (Figure 1a). There are between three to five decapeptide repeats in KAP1-1 [41], whereas KAP1-2, KAP1-3 and KAP1-4 have three, two and five repeats, respectively [29,42,43]. There is an apparent loss of a five residue C-terminus tail in KAP1-2, compared to the other KAP1 proteins [29] and KAP1-1 and KAP1-4 are acidic KAPs, while KAP1-2 and KAP1-3 are neutral (Table 1).

**KAP2**: KAP2 is a HS-KAP family. Five family members have been described in humans [40], but only two protein sequences (BIIIA3A and BIIIA3) have been reported in sheep (Figure 1b). These ovine sequences share 95% homology.

BIIIA3A is referred to as the orthologue of human KAP2-1 [8], although the same human sequence was previously called KAP2.12 [5]. BIIIA3 is identical to the predicated amino acid sequence of a partial DNA sequence referred to as KAP2-3 (GenBank U60024). To date no ovine KAP2-2 gene has not been reported. Given that the KAP2 family is conserved in humans, with over 97% sequence identity between the family members [8,40], it is likely that these two wool KAP2 sequences represent two members of the same family. The KAP2 family members possess several cysteine-rich pentameric repeat structures (CCXPX) and length differences are also observed among family members in both sheep and humans. The KAP2 proteins are weakly basic (Table 1).

**KAP3**: There are three major (originally designated as BIIIB2, BIIIB3 and BIIIB4) and one minor (originally called BIIIB1) proteins described for ovine KAP3 [44]. The three major proteins have been isolated and sequenced [9,11,12] and the gene sequences thought to encode the major proteins have also been identified [20]. These have been named KAP3-1, KAP3-2 and KAP3-3 representing BIIIB2, BIIIB3 and BIIIB4, respectively. Minor differences between the protein sequences and the gene sequences have been reported for each family member, which may reflect nucleotide variation in the genes, or alternatively sequencing errors. The BIIIB2 protein sequence (labelled as KAP3-1A) [9] appears to be different to the predicted amino acid sequence derived from translation of the BIIIB2 gene (labelled as KAP3-1B) [20], and shares only 94% homology at the amino acid sequence level. Interestingly, these two proteins would appear to have different pI values (Table 1), so whether these two BIIIB2 sequences represent different family members, or are variant forms of the same family member, requires further investigation. The human KAP3 family contains three functional genes and one pseudogene [8,40]. KAP3-2 and KAP3-3 are very similar to each other, but different to KAP3-1 (Figure 1c). Little repetitiveness of structure is seen in this family and the proteins are generally neutral.

**KAP4**: KAP4 is a UHS-KAP family. This family is reported to contain 11 members in humans [40], but only three members have been identified in sheep [5,22,31]. The ovine KAP4 proteins possess a repeat structure that covers a large portion of the middle section of the protein and consists of concatenates of a monocysteine- and dicysteine-containing pentameric repeat structure (Figure 1d). The proteins are weakly basic (Table 1).

**KAP5**: KAP5 belongs to the UHS group and is one of the largest KAP families, with 12 family members described in humans [8,37]. In sheep, three complete and one partial DNA sequences have been identified [4,21,23] and these have been designated KAP5-1, KAP5-2, KAP5-4 and KAP5-5. Sheep KAP5 proteins possess repeat structures that are either cysteine/serine-rich, or glycine-rich, and the repeat number varies between family members (Figure 1e). The proteins are weakly basic (Table 1).

**KAP6**: KAP6 is a HGT-KAP family. Three family members have been identified in the human genome [45]. Southern-hybridisation analysis revealed that there were potentially several family members in sheep and as many as twenty in mice [26]. Early research identified two ovine members designated KAP6-1 and KAP6-2 [18,26], but three additional members (designated KAP6-3, KAP6-4 and KAP6-5) have recently been identified in the sheep genome and submitted to GenBank [34]. The KAP6 proteins consist of repetitive units of glycine-tyrosine and glycine-tyrosine-glycine (Figure 1f), and they are weakly basic (Table 1).

**KAP7**: KAP7 belongs to a HGT-KAP family. This KAP family contains only one family member designated KAP7-1 [25]. There is high sequence similarity between the orthologs from sheep and humans, and the number of glycine-tyrosine repeat structures is small compared to the KAP6 family (Figure 1g). Ovine KAP7-1 is basic, with a pI value of 8.7 (Table 1).

**KAP8**: KAP8 is a HGT-KAP family. In sheep, it was thought to contain only one member [25], but another potential member called KAP8-2 [33] has recently been identified. In humans only one KAP8 gene has been identified [45]. KAP8 proteins exhibit high sequence similarity across species, but have fewer glycine-tyrosine repeat structures when compared to the KAP6 family (Figure 1h). KAP8-2 contains a high content (4.8 mol%) of aspartic acid and glutamic acid, which is not common in other HGT-KAPs, and hence it has a low pI value of 6.3 [33]. In sheep, KAP8-1 is weakly basic, while KAP8-2 is weakly acidic (Table 1).

**KAP11**: KAP11 is a HS-KAP family consisting of a single member categorised as KAP11-1 [27]. While KAP11-1 belongs to the HS group, the protein contains a low frequency of the “CCXP” motif that is common in the other cysteine-rich KAPs, and that is often found as part of longer repeat elements [5]. This motif is present in a single copy in KAP11-1 from sheep, cattle and humans, but it is absent in mouse KAP11-1. The KAP11-1 protein does not have a high content of glycine, but instead has proportionally more serine and threonine residues (Figure 1i). These two amino acids make up over 30 mol% of the residues in ovine KAP11-1 [27], but the biological function of having higher concentrations of serine and threonine is not known. As serine and threonine residues can be post-translationally phosphorylated, it may be associated with the phosphorylation of this protein. KAP11-1 is a weakly basic protein (Table 1).

**KAP13**: Belonging to the HS group, the KAP13 family has four members in humans [46], but only one member (KAP13-3) has been described in sheep [28]. Ovine KAP13-3 has a low sequence similarity to the human orthologues, and only a poorly conserved repeat structure is found (Figure 1j). Like KAP11-1, the ovine KAP13-3 protein contains a high content of serine and threonine, and many of these residues are potentially post-translationally modified. The ovine KAP13-3 protein contains more positively charged amino acids (arginine, lysine and histidine) and fewer negatively charged ones (aspartic acid and glutamic acid), and hence it has a pI value of 9.4 [28]. It is the most basic KAP protein identified to date in sheep (Table 1).

**KAP24**: There is only one member (KAP24-1) reported in sheep [30] and in humans [38]. While probably belonging to the HS group, the KAP24-1 protein contains a moderate level of cysteine, but a high content of serine and tyrosine. High tyrosine contents are typically not observed in other HS or UHS KAPs and additionally, the tyrosine in KAP24-1 is not part of the dimeric glycine-tyrosine repeats that are found in other high glycine-tyrosine KAPs [30]. Wool KAP24-1 is a basic protein (Table 1) and it has a low (~64%) sequence similarity to the human orthologue. The human KAP24-1 protein possesses a series of tandem decameric repeat structures at the C-terminal end [38], but similar structures are not obvious in the ovine KAP24-1 sequence (Figure 1k).

## 4. The Chromosomal Location of Wool KAP Genes

The detection of multiple KAP genes in single genomic clones from a variety of mammalian species, immediately suggested the grouping of KAP genes in the genome [19,21,47,48]. More recent bioinformatic analyses of genome sequences have confirmed this clustering in humans [40,45,49,50,51] and in a variety of others species [29,30,33,52].

**Figure 2:**
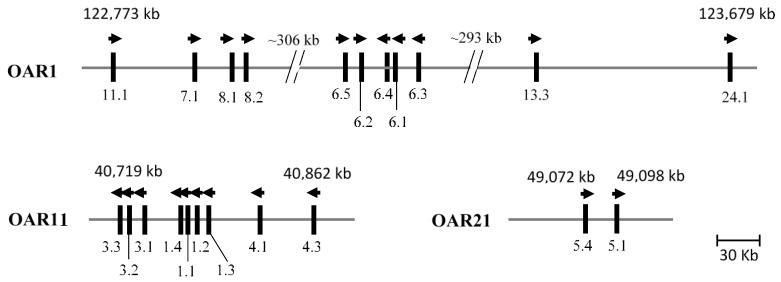
Clustering of the ovine KAP genes in three chromosome regions. The bolded vertical bars represent known KAP genes and the arrows indicate the direction of transcription. The numbers below these bars are the KAP gene names (*i.e.*, 11.1 refers to *KRTAP11-1*). The spacing of the genes is only approximate and is based on the Ovine Genome Sequence Assembly v4.0 sequence, where coordinates are given for the boundaries of the clusters, based on the first and last gene identified in the group.

The ovine KAP genes identified to date have been mapped to, or located on, three chromosomes. The KAP1-n, KAP3-n and KAP4-n genes are located on OAR11 [29,53,54]; the genes for the KAP6-KAP8, KAP11, KAP13 and KAP24 families are located on OAR1 [27,30,33,34,53]; and the KAP5-n genes are located on OAR21 [53,54] (Figure 2). The location of the KAP2-3 and KAP5-5 genes has not been determined, as only partial DNA sequences are available. The clustering and relative position of the various ovine KAP genes identified appears to correspond closely with that reported in humans [8,38].

## 5. Nucleotide Polymorphism within the KAP Genes

While the KAP genes (denoted as *KRTAP*s) have been identified in humans, only limited efforts have been made to fully understand the presence of nucleotide variation within those genes. To date, it has only been described for the KAP1 and KAP4 families and studies only undertaken in Caucasian and Japanese populations [8,35,36]. In contrast, variation has been extensively looked for in ovine KAP genes, including KAP1, KAP3, KAP5, KAP6-KAP8, KAP11, KAP13 and KAP24 genes. This likely reflects the importance of wool in the production of apparel and interior textile products.

In the KAP1 family, three variant sequences containing 13 SNPs and length variation (having between three to five repeats of a 30 bp sequence) have been described for *KRTAP1-1* [41,42], 11 variant sequences containing 10 SNPs have been described for *KRTAP1-2* [29,55], nine variant sequences containing 20 SNPs have been described for *KRTAP1-3* [41,42] and nine variant sequences containing 14 SNPs have been reported for *KRTAP1-4* [43].

The SNPs described in *KRTAP1-1* and *KRTAP1-4* are predominantly non-synomynous, whereas those decribed in *KRTAP1-2* and *KRTAP1-3* are predominantly synomynous. This appears to reflect the chromosomal locations of these genes, where *KRTAP1-1* and *KRTAP1-4* are close to each other on the chromosome and further removed from *KRTAP1-2* and *KRTAP1-3* [29]. It should however be noted that the sequence variants described for *KRTAP1-1* have been detected by PCR and agarose gel electrophoresis. This approach is inadequate for detecting SNPs and thus the varation described to date for *KRTAP1-1* may be an under-estimation of what actually exists in sheep. Further investigation using other nucleotide variation screening techniques is required to reveal the full extent of variation in this gene, if it exists.

In the KAP3 family, variation has only been revealed in *KRTAP3-2* [53]. Two variants of ovine *KRTAP3-2* detected by PCR-SSCP have been reported, but as no sequence information describing the nucleotide varation has been reported, the nature of the variation is currently unknown.

In the KAP5 family, five sequnec variants have been decribed for ovine *KRTAP5-4* [56]. There are six SNPs and one length polymorphism in this gene. Non-synonymous SNPs predominate and there is variation in copy number of a 30-bp repeat sequence encoding a cysteine-rich decapeptide ‘‘RPCCSQSSCC’’ in the C-terminal region. Either one, or two copies of the repeat sequence are present in each varaint.

In the KAP6 family, variaton has been investigated in all of the family members [32,34,57]. *KRTAP6-1* has been described to have three sequence variants that are comprised of three SNPs and a 57-bp insertion/deletion [32,57]. *KRTAP6-2* has six sequence variants containing five SNPs [34]. Five sequence variants are detected for *KRTAP6-3.* There are three SNPs and a 45-bp insertion/deletion observed for *KRTAP6-3* [34]. *KRTAP6-4* has been reported to have three SNPs resulting in three sequence variants [34]. There are six sequence variants described for *KRTAP6-3* that contain five SNPs and an 18-bp insertion/delection [34]. The SNPs found in *KRTAP6-3* and *KRTAP6-5* are predominantly non-synonymous, whereas synonymous SNPs predominate in *KRTAP6-1*, *KRTAP6-2* and *KRTAP6-4*. The nature of the polymorphism does not appear to coincide with the physical locations of the genes on the chromosome, but it is in some respects consistent with *KRTAP6-1*, *KRTAP6-2* and *KRTAP6-4* being located in the middle of a region that is flanked by *KRTAP6-5* and *KRTAP6-3*.

Variation in *KRTAP7-1* has been investigated by two groups [53,58]. McLaren *et al.* [53] reported two sequence variants using the restriction endonuclease *Bgl*II and four sequence variants using the restriction endonuclease *Msp*I in Southern hydridisation-Restriction Fragment Length Polymorphism (RFLP) analysis. No specific information on nucleotide sequence variation was given. Gong *et al.* [58] used PCR-SSCP to screen the entire coding region of *KRTAP7-1* and reported two sequence variants resulting from one non-synonymous SNP. This SNP is not in either a nominal *Bgl*II or *Msp*I restriction endonuclease recognition site. What is more, there are no *Bgl*II recognition sequences present, or that can be created by a single nucleotide substitution in the coding region of ovine *KRTAP7-1*. This suggests that these restriction endonuclease recognition sites were located outside of the coding region of the *KRTAP7-1* gene.

In the KAP8 family, varation has been investigated in both *KRTAP8-1* and *KRTAP8-2* [33,58]. *KRTAP8-1* has five sequnce variants containing four SNPs, whereas *KRTAP8-2* has only two sequnce variants containing one SNP. The majority of SNPs found in *KRTAP8-1* are synonymous [58], while the sole SNP found to date in *KRTAP8-2* is located 21 bp upstream of the nominal TATA box sequence [33].

Six and five sequnce variants are identified in ovine *KRTAP11-1* and *KRTAP13-3*, respectively [27,28]. There are five and four SNPs in these genes respectively, and four of the five SNPs in *KRTAP11-1* are synonymous, while three of the four SNPs in *KRTAP13-3* are non-synonymous.

Using PCR-stem loop conformational polymorphism (PCR-SLCP), Zhou *et al.* [59] reported four sequnce variants of ovine *KRTAP24-1*. Seven SNPs were described in ovine *KRTAP24-1* and four of them are non-synonymous.

In summary, variation has been found in all of the ovine KAP genes investigated to date. The apparent higher degree of variation found in sheep when compared to humans, is possibly due to a greater number of individuals having been screened. Genetic variation in human KAP genes could therefore potentially be significantly higher than previously thought based on comparison with ovine KAP genes, although this remains to be revealed.

## 6. The Expression of KAPs

In humans, all the KAP genes that have been identified to date are expressed in the hair follicle, with the exception of families 16, 22, 25 and 27 [8,38,39]. These KAP genes exhibit a uniform hair follicle expression pattern that is characteristic of the chromosome domain in which these gene are located. The exception is for the KAP genes on chromosome domain 21q22.1 and a single KAP gene (*KRTAP17-1*) on chromosome 17q21.2 [8]. The detailed expression of human KAP genes has been reviewed [8] and will not be described here.

**Figure 3:**
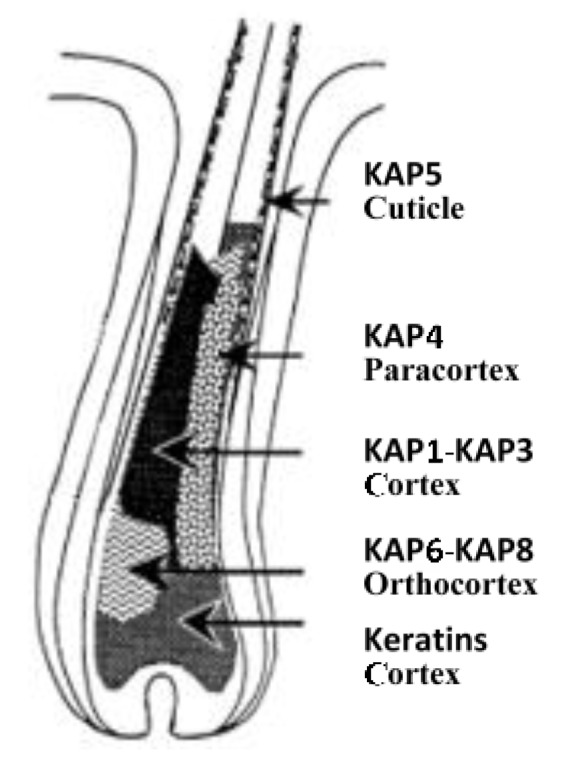
Schematic representation of the sites of KAP gene expression in the wool follicle. Modified from Powell and Rogers [5].

The expression of ovine KAPs is not well understood. Of the ovine KAP families investigated, the HGT-KAPs (KAP6, KAP7 and KAP8) are the first expressed in growing wool follicles and appear in the orthocortical cells [5,31]. The HS-KAPs (KAP1, KAP2 and KAP3) are initially expressed in the orthocortical cells, but soon after this are expressed in all cortical cells [5]. The UHS-KAP4 family is expressed later in follicle growth and only in paracortical cells [22,31]. The UHS-KAP5 family is expressed during cuticle differentiation [21,23]. A schematic summary of wool KAP expression is shown in Figure 3.

The expression of the KAP genes in sheep is consistent with that reported in humans, but some differences are observed. The most notable is the refined localisation of expression zones observed in wool follicles. For example, human KAP1 to KAP4, KAP6 and KAP7 are all expressed in the hair cortex [8], but in the wool follicle, KAP6 and KAP7 are only expressed in the orthocortex, and the expression of KAP4 is restricted to the paracortex [5,22,34]. While KAP1 and KAP2 are expressed across the entire cortex of wool follicles, their expression starts in the orthocortical half [5]. Such a well-defined bilateral expression pattern is not found in human hair follicles. In humans, KAP8 is expressed earlier than KAP6 and KAP7 [8], but they appear to be co-expressed in the wool follicle [5].

The weak expression of KAP6 in human hair [8] is of interest as it contrasts the high level of expression of KAP6 in the wool follicle. It should however be noted that the level of expression reported for the human KAP genes was based on the quantitation of mRNA level and not on the amount of protein observed in fibre [45]. It has been observed that there is only a moderate correlation between mRNA transcript levels and protein translation levels [60], and that the amount of mRNA present in a cell or tissue can be a poor predictor of protein levels, especially for genes with low mRNA expression. Equally, it has been reported that for some genes with similar levels of mRNA expression, the protein levels can vary 20- to 30-fold in difference [61].

## 7. KAP Genes and Wool Traits

There have been a number of studies describing associations between wool traits and variation in KAP genes.

Wool crimp is thought to be affected by the composition of KAPs in the wool fibre. Felting lustre (FL) mutant wool has a very low relative abundance of HGT-KAP proteins in the fibre itself [62], but the genes encoding the HGT-KAPs are present [63]. This suggests a reduction or failure of transcription or translation of the HGT-KAPs genes in FL mutant wool. Research suggested originally that the FL mutation was inherited in a single autosomal dominant fashion [64], which was later confirmed by Blair [65]. However, examination of transcript prevalence, suggests that the KAP6-1, KAP7-1 and KAP8-1 genes are down-regulated, while the KAP2-12 and KAP4-2 genes are up-regulated in mutant follicles [66]. In the FL mutant follicles, there is only one type of cortical cell present (paracortical) and the orthocortical cell type where the HGT-KAPs are usually expressed appear to be absent [66]. A recent report by Wang *et al.* [67] describes an OAR11 QTL for crimp that is approximately 30 MB from the known KAP genes on that chromosome.

Parson *et al.* [68] reported that variation in a KAP6 gene was associated with Mean Fibre Diameter (MFD) in medium wool Peppin Merinos. The KAP6 gene maps to ovine chromosome 1 [69] in a chromosome region where Beh *et al.* [70] detected a QTL affecting MFD in medium wool Merinos. On this chromosome, Roldan *et al.* [71] did not find any QTL for MFD, but instead they found a QTL for other wool traits including curvature and wool yield. Allain *et al.* [72] also detected a QTL on chromosome 1 for the “objectionable fibre content” (being defined as a large medulated fibres with a latticed medulla deficient in sulphur) in backcross Sarda × Lacaune sheep. Wang *et al.* [67] describe two chromosome 1 QTLs for fibre diameter, but they are at least 100 MB from the known OAR1 KAP genes.

Recently, variation in *KRTAP6-1* was found to be associated with variation in wool fibre diameter associated traits, and a 57-bp deletion in the gene was associated with the occurrence of coarser wool with a greater Fibre Diameter Standard Deviation (FDSD), a greater Coefficient of Variation of Fibre Diameter (CVFD) and increased Prickle Factor (percentage of fibres over 30 microns; PF) [57].

On ovine chromosome 11, Rogers *et al.* [73] reported a putative QTL for wool staple strength in the region spanning *KRTAP1-1*, *KRTAP1-3* and *KRT33A* (*KRT1-2*) in Romney sheep. On this chromosome, Roldan *et al.* [71] detected a QTL for wool weight, staple strength and CVFD. Itenge-Mweza [74] reported an association between variation in *KRTAP1-1* and wool yield in one half-sib family and with Mean Staple Length (MSL) and wool brightness in another half-sib family. An association between variation in *KRTAP1-2* and Greasy Fleece Weight (GFW) and Clean Fleece Weight (CFW) was revealed in a recent study, suggesting *KRTAP1-2* mainly affects wool weights [55]. This is in agreement with a study in goats reporting that *KRTAP1-1* which is near *KRTAP1-2*, affects cashmere yield [75].

Finally, Raadsma *et al.* [76] report that OAR1 contains a QTL for wool pigmentation. The QTL (LOD score 2.5) is located near markers MAF64 and CSSM4 (192-256 cM) on chromosome 1 and while these are proximal to the KAP genes on that chromosome, it would be difficult to mount an argument of how the *KRTAP*s affect wool pigmentation specifically. This is not to say they do not affect pigmentation.

These findings suggest that KAP genes have a significant effect on wool traits, with further investigation required to fully explore this potential. While Wang *et al.* [67] found SNPs that might be associated with specific wool traits as described above, another more recent study using a SNP chip across 84 predicted KAP and keratin genes (59 SNPs were tested), did not reveal any association with fleece weight, wool fibre diameter and fibre curvature [77]. A SNP chip of these size may not have sufficient resolving power to precisely identify variation in ovine KAP genes, as many of the genes are highly polymorphic, having multiple variant forms as a consequence of containing many SNPs and insertions/deletions.

Given that KAP genes are clustered and that all the ovine KAP genes described to date are polymorphic and potentially expressed in the wool fibre, it will undoubtedly be difficult to unravel and determine the effects of individual genes on wool traits. However, the discovery of associations between variation in KAP genes and fibre traits, may ultimately allow for the future development of gene-markers for improving wool quality and, thus, enable the production of wool with specifications and performance more closely matched to targeted end-uses.

## Figures and Tables

**Table 1:** Ovine keratin-associated proteins (KAP) protein and gene sequences identified.

KAP Family	Category	KAP Member	Old Name	Acidity/Basicity (Isoelectronic Point)	Sequence Type	Sequence Accession Number	Reference
KAP1	HS	KAP1-1	B2A	Acidic (5.9)	Protein *	P02438	[13]
					DNA	X01610 ^#^	[19]
		KAP1-2	B2B	Neutral (7.0)	Protein	P02439	[15]
					DNA	HQ897973 ^#^	[29]
		KAP1-3	B2C	Neutral (7.3)	Protein	711148A	[14]
					DNA	X02925 ^#^	[19]
		KAP1-4	B2D	Acidic (5.9)	DNA	X01610 ^#^	[19]
KAP2	HS	KAP2-1	BIIIA3A, KAP2.12	Basic (8.1)	Protein	P02443	[16]
		KAP2-3	BIIIA3	Basic (8.1)	Protein	P02441	[16]
					DNA *	U60024	Unpublished
KAP3	HS	KAP3-1A	BIIIB2	Neutral (7.0)	Protein	P02446	[9]
		KAP3-1B	BIIIB2	Neutral (7.4)	DNA	M21099	[20]
		KAP3-2	BIIIB3	Neutral (7.0)	Protein	P02444	[11]
					DNA *	M21100	[20]
		KAP3-3	BIIIB4	Neutral (7.0)	Protein	P02445	[12]
					DNA	M21103	[20]
KAP4	UHS	KAP4-1		ND	DNA *	X73462	[22]
		KAP4-2		Basic (8.1)	Protein		[5]
		KAP4-3		Basic (8.2)	DNA	EU239778	[31]
KAP5	UHS	KAP5-1		Basic (7.9)	DNA	X55294	[21]
		KAP5-2		ND	DNA *		[4]
		KAP5-4		Basic (7.9)	DNA	X73434 ^#^	[23]
		KAP5-5		ND	DNA *	X73435	[23]
KAP6	HGT	KAP6-1	HGT type II	Basic (8.3)	DNA	M95719 ^#^	[22]
		KAP6-2	HGT type II	Basic (8.2)	Protein *		[18]
					DNA	KT725827 ^#^	[34]
		KAP6-3		Basic (8.4)	DNA	KT725833 ^#^	[34]
		KAP6-4		Basic (8.2)	DNA	KT725838 ^#^	[34]
		KAP6-5		Basic (8.1)	DNA	KT725841 ^#^	[34]
KAP7	HGT	KAP7-1	HGT-C2	Basic (8.7)	DNA	X05638	[25]
KAP8	HGT	KAP8-1	HGT-F	Basic (8.3)	Protein	P02448	[17]
					DNA	X05639 ^#^	[25]
		KAP8-2		Acid (6.3)	DNA	KF220646 ^#^	[33]
KAP11	HS	KAP11-1		Basic (8.0)	DNA	HQ595347 ^#^	[27]
KAP13	HS	KAP13-3		Basic (9.4)	DNA	JN377429 ^#^	[28]
KAP24	HS	KAP24-1		Basic (8.5)	DNA	JX112014 ^#^	[30]

ND -Not determined as only a partial sequence is available. Partial sequences are indicated with *. All sequence accession numbers refer to the GenBank, except for 711148A which refers to the EMBL. When multiple DNA sequences have been identified, only one for each family member is listed and indicated by #.
